# Supplementary material for: Energy Transfer and Restructuring in Amorphous Solid Water upon Consecutive Irradiation
Source: J Phys Chem A. 2022 Nov 16;126(47):8859–70. doi: 10.1021/acs.jpca.2c06314 (PMC9720723; doi:10.1021/acs.jpca.2c06314)
Supplement: Supplementary file 1 — jp2c06314_si_001.pdf [file jp2c06314_si_001.pdf]

Supporting Information: Energy Transfer and Restructuring in Amorphous Solid Water upon Consecutive Irradiation

Herma M. Cuppen,[*,†,‡] Jennifer A. Noble,[*,¶,§] Stephane Coussan,[¶] Britta Redlich,[‖] and Sergio Ioppolo[*,⊥,#]

†*Radboud University, Institute for Molecules and Materials, Nijmegen 6525 AJ, The Netherlands*

‡*Van't Hoff Institute for Molecular Sciences, University of Amsterdam, Amsterdam 1098 XH, The Netherlands*

¶*CNRS, Aix-Marseille Univ, PIIM, Marseille 13397, France*

§*School of Physical Sciences, University of Kent, Canterbury CT2 7NH, U.K.*

‖*FELIX Laboratory, Radboud University, Nijmegen 6525 ED, The Netherlands*

⊥*Center for Interstellar Catalysis, Department of Physics and Astronomy, Aarhus University, Ny Munkegade 120, Aarhus C 8000, Denmark*

#*School of Electronic Engineering and Computer Science, Queen Mary University of London, London E1 4NS, U.K.*

E-mail: h.cuppen@science.ru.nl; jennifer.noble@univ-amu.fr; s.ioppolo@phys.au.dk

Tables S1 and S2 show the experimental settings for the different irradiation experiments for pASW and cASW, respectively. The irradiation order indicates consecutive irradiations at the same ice spot. Experiments on pristine ice have irradiation order "1". The power is measured at the FEL initial station prior to any gold mirror steering of the beam into the bays

area and before the CsI window separating LISA from the rest of the beamline. Therefore the actual power reaching the ice is estimated to be roughly half the values reported in the tables.

Table S1: Irradiation experiments on pASW (June 2018)

Irradiation order	wavelength (μm)	duration (minutes)	power (mJ)	notes
		"blue to red" series		
1	2.7	5	9	
2	2.8	5	12	
3	2.9	5	11	
4	3.0	5	14	
5	3.1	5	14	
6	3.2	1.5	12	FELIX was unstable
7	3.25	5	6	
		"red to blue" series		
1	3.25	2	6	
2	3.2	2	10	
3	3.1	2	13	
4	3.0	2	13	
5	2.9	2	11	
6	2.8	2	9	
7	2.7	2	8	
		Single irradiation		
1	3.1	5	12.5	

Table S2: Irradiation experiments on cASW (August 2018)

Irradiation number	wavelength (μm)	duration (minutes)	power (mJ)	notes
"blue to red" series				
1	2.7	3	7	
2	2.8	3	11	
3	2.9	3	14	
4	3.0	3	13	
5	3.1	3	15	
6	3.2	3	15	
7	3.25	3	15	
"red to blue" series				
1	3.25	3	15	
2	3.2	3	20	
3	3.1	3	20	
4	3.0	3	14	
5	2.9	3	14	
6	2.8	3	14	
7	2.7	3	14	
